# Measuring Moderate-Intensity Exercise with the Apple Watch: Validation Study

**DOI:** 10.2196/cardio.8574

**Published:** 2018-02-28

**Authors:** Grant Abt, James Bray, Amanda Clare Benson

**Affiliations:** ^1^ School of Life Sciences The University of Hull Kingston upon Hull United Kingdom; ^2^ Department of Health and Medical Sciences Swinburne University of Technology Melbourne Australia

**Keywords:** smartwatch, wearables, technology, physical activity, cardiovascular health, Apple Watch

## Abstract

**Background:**

Moderate fitness levels and habitual exercise have a protective effect for cardiovascular disease, stroke, type 2 diabetes, and all-cause mortality. The Apple Watch displays exercise completed at an intensity of a brisk walk or above using a green “exercise” ring. However, it is unknown if the exercise ring accurately represents an exercise intensity comparable to that defined as moderate-intensity. In order for health professionals to prescribe exercise intensity with confidence, consumer wearable devices need to be accurate and precise if they are to be used as part of a personalized medicine approach to disease management.

**Objective:**

The aim of this study was to examine the validity and reliability of the Apple Watch for measuring moderate-intensity exercise, as defined as 40-59% oxygen consumption reserve (VO_2_R).

**Methods:**

Twenty recreationally active participants completed resting oxygen consumption (VO_2_rest) and maximal oxygen consumption (VO_2_ max) tests prior to a series of 5-minute bouts of treadmill walking at increasing speed while wearing an Apple Watch on both wrists, and with oxygen consumption measured continuously. Five-minute exercise bouts were added until the Apple Watch advanced the green “exercise” ring by 5 minutes (defined as the treadmill inflection speed). Validity was examined using a one-sample t-test, with interdevice and intradevice reliability reported as the standardized typical error and intraclass correlation.

**Results:**

The mean %VO_2_R at the treadmill inflection speed was 30% (SD 7) for both Apple Watches. There was a large underestimation of moderate-intensity exercise (left hand: mean difference = -10% [95% CI -14 to -7], d=-1.4; right hand: mean difference = -10% [95% CI -13 to -7], d=-1.5) when compared to the criterion of 40% VO_2_R. Standardized typical errors for %VO_2_R at the treadmill inflection speed were small to moderate, with intraclass correlations higher within trials compared to between trials.

**Conclusions:**

The Apple Watch threshold for moderate-intensity exercise was lower than the criterion, which would lead to an overestimation of moderate-intensity exercise minutes completed throughout the day.

## Introduction

### Background

Physical activity is well documented as a beneficial intervention for the prevention and treatment of chronic disease, while physical inactivity is an independent risk factor for the development of lifestyle-related chronic diseases, such as cardiovascular disease [1,2]. To be considered “physically active” it is recommended that adults accumulate >150 minutes of moderate or 75 minutes of vigorous-intensity physical activity (MVPA) over the course of a week to provide them with substantial health benefits [3]. Given that the average adult expends approximately four metabolic equivalents walking at a moderate pace [4], they would therefore need to walk at this pace for 125 minutes/week, or 30 minutes on most days of the week, to meet the minimum recommendations. Although these physical activity guidelines are strongly recommended by governments across the world [5-7], many people do not achieve them [8-11]. However, physical activity interventions that have incorporated technology-based support have shown promise in developing compliance to physical activity guidelines [12,13], possibly through reinforcement to develop habit-forming behaviors [14].

Although valid and reliable tools for measuring physical activity are available for researchers, such as the ActiGraph [15], it is the quantified-self movement [16] that has led to the increasing popularity of consumer wearable technology, with estimates indicating sales of over 200 million by 2021, including the Apple Watch [17]. Having contemporary instruments to integrate into exercise prescription and physical activity promotion that fit into people’s lifestyles is imperative. Although evidence on the validity and reliability of modern wearable devices with integrated screens to measure exercise and physical activity is increasing, no study has examined the accuracy of the Apple Watch for measuring MVPA.

Apple is one of the world’s most valuable companies (by market value) [18] and has changed a number of industries through disruptive devices (eg, iPod, iPhone). The Apple Watch, which was released in 2015, has reportedly become the highest selling wearable/smartwatch to date, with more than 12 million units reported to have been sold [19,20]. The widespread use of the Apple Watch would therefore allow physical activity interventions to reach a large proportion of the population. Additionally, the Apple Watch provides potential for data to be collected from, and returned to, the individual, to provide immediate individualized feedback via the wearable’s screen to promote behavior change and be shared with others, such as a clinician. However, Apple provides little detail on how the Apple Watch measures “exercise.” It appears that the built-in accelerometer is mostly used to measure physical activity throughout the day [21,22], although heart rate measurement is used periodically when walking [22]. Daily physical activity data is displayed within the “Activity” app, with a visual representation of the accumulated duration of “exercise” displayed as a green ring on the screen of the watch. Given that Apple refers to this as the “exercise” ring and that 30 minutes is the daily goal [23], it is reasonable to suggest that Apple views this as representative of the daily goal of 30 minutes of MVPA for adults, as recommended by numerous guidelines [5-7]. Although Apple states that, “every full minute of movement that equals or exceeds the intensity of a brisk walk counts toward your daily exercise goal” [23], it is not clear how this measure of MVPA compares to the criterion measure of moderate-intensity exercise, oxygen consumption reserve (VO_2_R: [VO_2_max - VO_2_rest] x exercise intensity + VO_2_rest).

### Objective of This Study

Given the scale of smartwatch use around the world and the increasing attention on personalized medicine, the validity of the Apple Watch for measuring moderate-intensity exercise is important to examine. Establishing the validity of the Apple Watch would ensure that individuals are able to measure their own MVPA accurately and that health professionals have confidence in the data that their clients are sharing with them. Establishing the intradevice and interdevice reliability of the Apple Watch is also important so that daily measures can be compared, and that user preference for wearing the Watch on the left or right wrist does not introduce bias. Therefore, the aim of this study was to examine the validity and reliability of the Apple Watch for quantifying moderate-intensity exercise compared with directly measured VO_2_R.

## Methods

### Study Design and Participants

The study used a repeated measures design with each participant completing two main trials. Prior to the main trials, maximal oxygen consumption (VO_2_max) and resting oxygen consumption (VO_2_rest) were measured in all participants. The study was approved by the Department of Sport, Health and Exercise Science Ethics Committee (approval number 1516076) at the University of Hull. Given the paucity of data on the Apple Watch at the time of study commencement, the sample size was estimated based on a previous study [24].

### Recruitment

Participants were recruited from the University of Hull and local community via written promotional material or personal communication. Inclusion criteria stated that participants were aged between 18 and 50 years, and exclusion criteria were: (1) men and women classified as moderate or high-risk according to the American College of Sports Medicine (ACSM) risk classification criteria, (2) those unable to walk on a motorized treadmill, (3) current smokers, (4) BMI >30, and (5) those currently taking medication that alters the heart rate in response to exercise (eg, beta blockers). Inclusion and exclusion criteria were established with the aim of recruiting low-risk individuals, based on the ACSM risk classification criteria used at the commencement of the study.

### Data Collection

#### Anthropometric

Participants were asked if they had voided before attending the session; if not, they were instructed to do so. Participants were then instructed to remove all clothing and nude body mass was measured to the nearest 0.1 kg using digital scales (WB-100MA Mark 3, Tanita Corporation, Tokyo, Japan). The mean of two measurements was used for further analysis. Stretch stature was measured using a wall-mounted stadiometer (Holtain Ltd, Dyfed, Wales, UK) and according to the methods of the International Society for the Advancement of Kinanthropometry [25].

#### Familiarization

Following medical screening and admission to the study, participants were familiarized with the tasks required of them during the main trials. This familiarization session consisted of the participant practicing “hopping on” and “hopping off” the treadmill, as well as walking at a number of dedicated speeds. This familiarization procedure was repeated at three of the speeds used in the main trials (ie, 3, 4.5, 6 km/hour).

#### Resting Oxygen Consumption

VO_2_rest was measured 30 minutes prior to, and in the same session, as VO_2_ max in a temperature-controlled laboratory. Participants lay supine on a bed with their head on a pillow for approximately 22 minutes. Oxygen consumption was measured continuously from expired air using a breath-by-breath online gas analysis system (Cortex Metalyzer 3B, GmbH, Germany). The analyzer was calibrated prior to each test using room air and known gas concentrations of oxygen and carbon dioxide. Volume was calibrated using a 3-liter syringe.

During the 22 minutes of measurement, the laboratory lights were turned off and all other laboratory activity was stopped. Prior to commencement of the measurement period, participants were instructed to relax as much as possible but to avoid going to sleep; they were not permitted to close their eyes. For analysis of the data, we discarded the first 10 minutes of data to allow for habituation and the last 2 minutes of data to allow for expectation effects. The mean of the remaining 10 minutes of data was taken as the VO_2_rest. Although we are not aware of any standardized method for measuring VO_2_rest for the purpose of calculating VO_2_R, we developed our method based on that reported by Miller et al [26].

#### Maximal Oxygen Consumption

Maximal oxygen consumption was determined on a motorized treadmill (h/p/cosmos, Pulsar, Nussdorf-Traunstein, Germany) using an incremental protocol that commenced at 3 km/hour and a 1% gradient and increased 0.5 km/hour in speed every 30 seconds until volitional fatigue. Oxygen consumption was measured continuously from expired air using the same breath-by-breath system as described above for VO_2_rest.

#### Exercise Protocol

For the 24 hours prior to each trial, participants were instructed to avoid exercise and maintain their normal diet, and for three hours prior to each trial avoid food and caffeinated drinks. On two separate occasions (mean 6 days apart, SD 3) participants completed a series of 5-minute bouts of walking on a treadmill at a gradient of 1%. Each bout was followed by 5-minutes of seated rest. On both occasions, the first 5-minute walking bout was conducted at 3 km/hour, with the treadmill speed increased for each successive 5-minute bout by 0.5 km/hour (ie, 3.5, 4). Exercise bouts were continued until at least 6 km/hour was completed, and until the Apple Watch indicated that all 5-minutes of that bout was at a sufficient intensity to accumulate 5-minutes of the green “exercise” ring, as displayed within the Activity app. The treadmill speed at which this occurred was defined as the “treadmill inflection speed.” During each 5-minute period of exercise, oxygen consumption and heart rate were recorded by an online gas analysis system (as described previously), a Polar chest strap (Polar T31, Polar Electro, OY, Finland), and an Apple Watch (described below) worn on each wrist. During each 5-minute period of exercise, participants were instructed to maintain their normal gait and were not permitted to hold the treadmill handrails. Immediately at the cessation of each 5-minute exercise period, participants were instructed to grasp the treadmill handrails and straddle the treadmill belt. Once the treadmill belt was stationary, a chair was placed on the treadmill and the participant was instructed to sit. During the recovery period participants were required to sit motionless with each hand resting on the treadmill handrail. This was done to ensure that no activity during the recovery period contributed to the green “exercise” ring. Five minutes of seated rest was provided to enable each Apple Watch to update the green “exercise” ring. In pilot testing it was observed that the Apple Watch completed its update within a maximum of 5 minutes of rest following exercise. The mean oxygen consumption of the last three minutes at the treadmill inflection speed for each watch was used for later analysis.

Two first-generation (Series 0) Apple Watches running watchOS 2.0.1 were used to estimate moderate-intensity exercise. Each Apple Watch was paired to an iPhone 6 running iOS 9.1. Following each 5-minute rest period the number of “exercise” minutes, as measured by each of the Apple Watches, was manually recorded from the Activity app.

Moderate-intensity exercise is defined by the ACSM as that which elicits an oxygen consumption of between 40% and 59% of VO_2_R. By rearranging the equation provided by the ACSM (see below) [3] and substituting target volume of oxygen (VO_2_) for the measured oxygen consumption at the treadmill inflection speed, the percentage of VO_2_R at the treadmill inflection speed (exercise intensity in the equation) can be calculated: Target VO_2_= (VO_2_max - VO_2_rest) x exercise intensity + VO_2_rest.

### Statistical Analyses

Data were checked for normality using the Shapiro-Wilk test and graphical methods, and were found to be plausible. The VO_2_R during Trial 2 was used to assess the validity of the Apple Watch for measuring moderate-intensity exercise, with VO_2_R during both Trial 1 and 2 used to assess the interdevice and intradevice reliability. A one-sample t-test was used to test if the mean %VO_2_R at the treadmill inflection speed for each Apple Watch was different from 40%, which is the lower limit of moderate-intensity exercise. A custom-designed Excel spreadsheet was used to examine differences between left and right Apple Watches for treadmill inflection speed and %VO_2_R [27]. Pearson product-moment correlation was used to assess the association between VO_2_max and the %VO_2_R at the treadmill inflection speed. Based on the linear association between VO_2_max and %VO_2_R, linear regression was used to estimate the VO_2_ max and treadmill speed required for the Apple Watch to accurately measure moderate-intensity exercise. Interdevice and intradevice reliability is reported as the standardized typical error and intraclass correlation. Standardized typical error was doubled prior to assessing its magnitude [28]. Standardized effect size is reported as Cohen’s *d* using the between-subject pooled SD as the denominator. The scale of magnitudes used to evaluate Cohen’s *d* was: 0-0.19 *trivial*; 0.2-0.59 *small*; 0.6-1.19 *moderate*; 1.2-1.99 *large*; >2.0 *very large* [28]. Uncertainty in the population estimates are reported as 95% CIs.

## Results

Twenty (10 male, 10 female) recreationally active participants (mean age 32 years [SD 10]; body mass 71.4 kg [SD 14.2]; stature 174.5 cm [SD 7.2]) provided written informed consent to undertake a maximal exercise test and the research exercise protocol. Participants had their cardiovascular risk assessed using the ACSM risk classification guidelines [3], with all participants classified as low risk.

**Figure 1:**
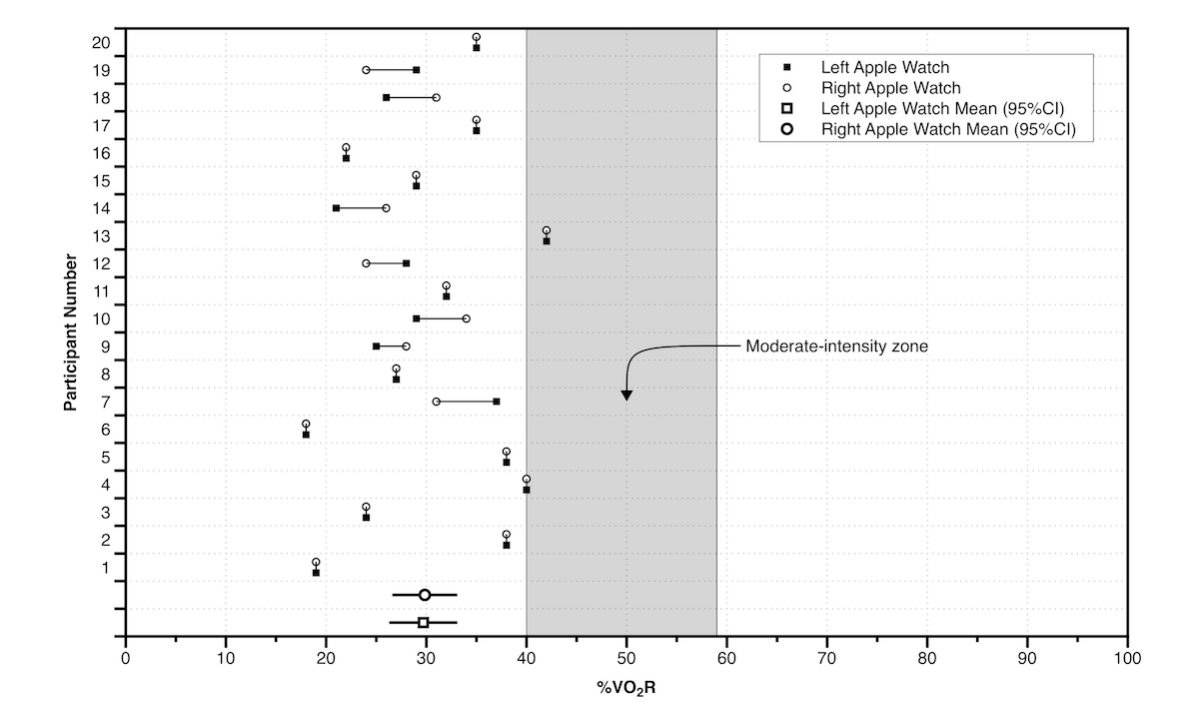
The oxygen consumption reserve (%VO_2_R) at the treadmill inflection speed for each participant and each watch compared with the moderate-intensity zone.

The mean VO_2_max and VO_2_rest, as measured using the online gas analysis system, were 45 mL/kg/minute (SD 10) and 3.4 mL/kg/minute (SD 0.6), respectively. The mean treadmill “inflection” speeds that were required to advance the Apple Watch green exercise ring by 5 minutes were 5.6 km/hour (SD 0.5) and 5.6 km/hour (SD 0.5) for the left and right Apple Watches, respectively (mean difference: 0 km/hour [95% CI -0.1 to 0.2], *d*=0.05 [trivial]). The mean %VO_2_R at the treadmill inflection speed for the left and right Apple Watches were 30% (SD 7) and 30% (SD 7), respectively (mean difference: 0% [95% CI -1 to 2], *d*=0.02 [trivial]). When compared to the criterion threshold of 40% VO_2_R, this represents a large underestimation in the ability of the Apple Watch to measure moderate-intensity exercise (left: mean difference = -10 [95% CI -14 to -7], *d*=-1.4 [large]; right: mean difference = -10 [95% CI -13 to -7], *d*=-1.5 [large]). The %VO_2_R at the treadmill inflection speed for each participant and each watch is displayed, together with the mean and 95% CI for both watches, in Figure 1.

**Figure 2:**
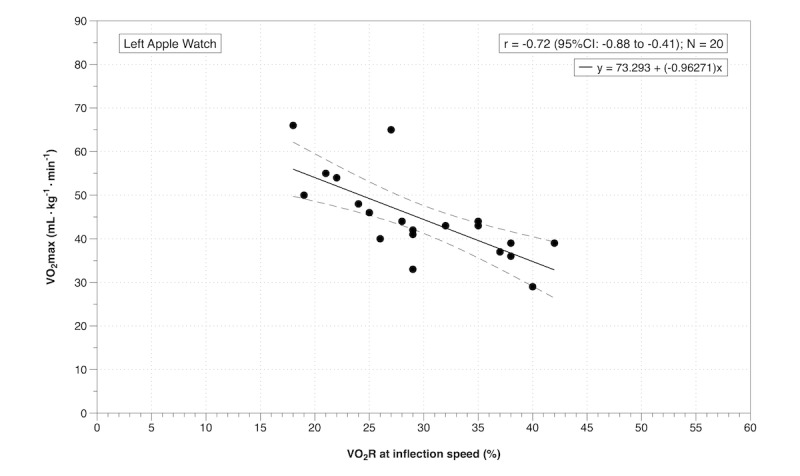
Pearson correlation between oxygen consumption reserve (%VO_2_R) at the treadmill inflection speed and maximal oxygen consumption (VO_2_max) for the left Apple Watch.

**Figure 3:**
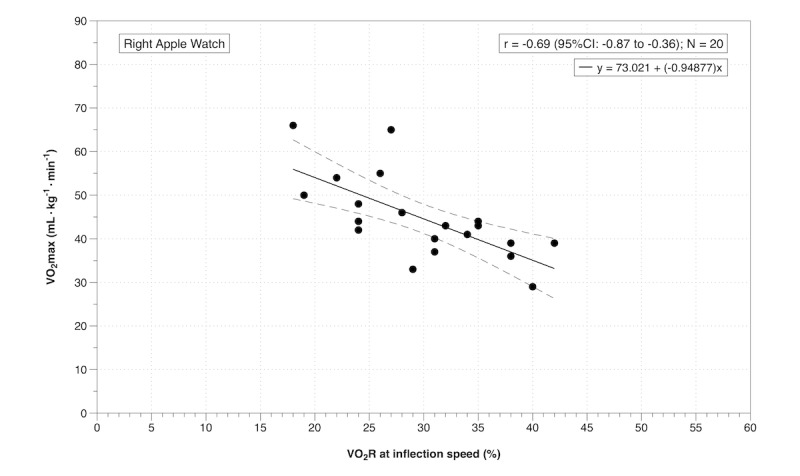
Pearson correlation between oxygen consumption reserve (%VO_2_R) at the treadmill inflection speed and maximal oxygen consumption (VO_2_max) for the right Apple Watch.

There was a very large negative correlation between %VO_2_R at the treadmill inflection speed and VO_2_max for the left Apple Watch (Figure 2), and a large negative correlation between %VO_2_R at the treadmill inflection speed and VO_2_max for the right Apple Watch (Figure 3). Participants with a higher VO_2_max were exercising at a lower percentage of their VO_2_R at the treadmill inflection speed (Figures 2 and 3). For the Apple Watch to accurately measure moderate-intensity exercise (40-59% VO_2_R), based on the regression equation for the left Watch, the user would need a VO_2_max between 16 (95% CI -3 to 36) and 35 (95% CI 19 to 50) mL/kg/minute. Based on within-participant linear regression analyses using the VO_2_R at each speed, we estimate that to achieve 40% of VO_2_R, participants would need to walk at a mean treadmill speed of 7.7 km/hour (95% CI 6.7 to 8.6) at 1% incline; which is 2.1 km/hour faster than that predicted by the Apple Watch. Only one participant had a comparable treadmill speed estimate for 40% VO_2_R between the Apple Watch and the online gas analysis system. Interdevice and intradevice reliability statistics for both treadmill speed and VO_2_R are displayed in Table 1.

**Table 1:** Intradevice and interdevice reliability for both treadmill speed and oxygen consumption reserve (VO_2_R) for left and right Apple Watches.

Parameter		Trial 1 Left vs Trial 1 Right	Trial 2 Left vs Trial 2 Right	Trial 1 Left vs Trial 2 Left	Trial 1 Right vs Trial 2 Right
**Speed**					
	Standardized typical error (95% CI)	0.41 (0.31 to 0.60)	0.54 (0.41 to 0.79)	0.53 (0.40 to 0.79)	0.64 (0.49 to 0.92)
	Qualitative interpretation	Moderate	Moderate	Moderate	Large
	Intraclass correlation (95% CI)	0.87 (0.70 to 0.95)	0.79 (0.55 to 0.91)	0.80 (0.55 to 0.92)	0.73 (0.45 to 0.88)
**VO_2_R**					
	Standardized typical error (95% CI)	0.2 (0.15 to 0.29)	0.33 (0.25 to 0.50)	0.48 (0.36 to 0.71)	0.37 (0.28 to 0.54)
	Qualitative interpretation	Small	Moderate	Moderate	Moderate
	Intraclass correlation (95% CI)	0.97 (0.92 to 0.99)	0.91 (0.78 to 0.96)	0.83 (0.61 to 0.93)	0.89 (0.75 to 0.95)

## Discussion

### Principal Findings

This is the first study to investigate the validity and reliability of the Apple Watch for measuring moderate-intensity exercise (the green “exercise” ring) compared to the criterion measure of VO_2_R. The Apple Watch largely underestimated the walking speed required to elicit the lower bound of moderate-intensity exercise (40% VO_2_R), which is an important part of individualized [3] and population-based [29] exercise prescription guidelines. The standardized typical error, a measure of the “typical” test-retest variability presented in units of SD [28], was small to moderate with no mean difference between the left and right watches for the mean treadmill inflection speed or %VO_2_R for the lower limit of moderate-intensity exercise [3].

For less fit individuals (<35 mL/kg/minute), using the Apple Watch to monitor moderate-intensity exercise is more likely to have the expected and desired improvements to fitness or clinical outcomes than for fitter individuals. However, for those that are fitter (>35 mL/kg/minute) it appears from our data that individuals would not meet the expectations of the lower end (40% VO_2_R) of the moderate-intensity range [3], with the Apple Watch underestimating the threshold for moderate-intensity exercise, and therefore overestimating the number of minutes of moderate-intensity exercise an individual had completed. Thompson et al [30] recently came to a similar conclusion, reporting that commercially-available wearable devices for self-monitoring of physical activity overestimate MVPA by a factor of 5-to-7-fold because they capture all physical activity, including normal moderate-to-vigorous lifestyle activities. However, other recent studies have reported that Fitbit devices (Fitbit One and Fitbit Flex) underestimate MVPA [31,32], although in these studies the wearable device was compared against another wearable device (ActiGraph), not direct laboratory-measured moderate-intensity exercise as we have done in our study. In our study only two participants achieved a %VO_2_R within the moderate-intensity exercise zone (Figure 1). Apart from the impact this may have on the expected physiological adaptations and fitness, it also has potential implications for morbidity and mortality risk, as moderate fitness levels and habitual exercise have a protective effect for cardiovascular disease, stroke, type 2 diabetes, and all-cause mortality [33].

Measuring load in an individual is complicated with a variety of methods available to monitor internal and external load [34]. The Apple Watch does not substantially overestimate or underestimate heart rate [24] and has moderate interdevice variability of maximal heart rate when worn on each wrist [35]. Therefore, using a continuous combination of internal (heart rate) and external (accelerometer) training load measures [34,36] rather than using the accelerometer plus periodic use of heart rate as is currently used, may improve the accuracy of the green exercise ring and provide a greater personalization of the data available to an individual. Given the wide range of VO_2_R responses observed at the treadmill speed that the Apple Watch determined to be “exercise” (<20% to >40%), and the variability between left and right Watches (Figure 1), it is clear that the Apple Watch needs to provide a more appropriate measure of the individualized response to exercise. This would not only enable more tailored feedback and exercise prescription, it would likely improve the physiological adaptations to any training and the associated cardiometabolic and musculoskeletal improvements for chronic disease prevention and treatment [33]. Better compliance to guidelines [13] and improved disease management and confidence [37] have been reported in healthy and chronic disease populations when technology-based support is incorporated. However, consumer and health professionals need to have confidence that any wearable device can both consistently and accurately measure the exercise intensity for individuals of all fitness levels.

The reliability data (Table 1) suggest that the reliability of VO_2_R at the point where the Apple Watch determines that “exercise” has started (the treadmill inflection speed) is better within trials (interdevice) than between trials (intradevice). This finding would suggest that physical activity measurements are more reliable within the same training session or activity compared to between different training sessions or activities. The implication is that health professionals can be confident that the ability of the Apple Watch to measure physical activity (as used in the current study) is not adversely affected by the wrist (left or right) on which an individual wore the watch within a given training session or activity.

### Strengths and Limitations

The main strength of our study is that this is the first investigation to examine the validity of the Apple Watch for measuring aspects of exercise related to the achievement of daily MVPA. The main limitation of this study is that we did not have direct access to the algorithms used by the Apple Watch for determining the exercise intensity at which the green “exercise” ring advances. Unfortunately, Apple does not publish these algorithms, probably due to commercial reasons. However, this is a limitation with most commercially-available wearable devices and is not restricted to the Apple Watch. A second limitation is that our data were derived from first generation Apple Watches. Although the method Apple uses to determine “exercise” using the Activity app has changed slightly with the periodic measurement of heart rate during walking [21,22], it is unclear how this would affect the validity of the Apple Watch for measuring MVPA. Further studies are required using the latest generation of Apple Watch for this to be determined. The exercise protocols used in our study were also constrained to linear walking on a treadmill. Movement patterns used by people outside of the laboratory while wearing an Apple Watch may result in different physiological responses and different determinations of “exercise” by the Apple Watch. Fourth, although our primary measure was VO_2_R, there are a number of factors that can influence the oxygen cost of exercise, such as economy [38,39]. For example, economy has been reported to be affected by age, such that older adults have less economy when walking compared to younger people [40]. The mean age of our participants was 32 years (SD 10), which places our participants midway between the young and old adults participating in the study of Martin et al [40]. Although factors such as economy and age should be taken into account when interpreting our results, the use of %VO_2_R as a relative measure of oxygen cost means that between-individual comparisons should still be meaningful.

### Conclusions

The Series 0 Apple Watch underestimates the threshold for moderate-intensity exercise compared to the criterion measure of VO_2_R, which would result in an overestimation of the amount of MVPA undertaken throughout the day. This effect is more pronounced in fitter individuals.

## Abbreviations

ACSM: American College of Sports Medicine
MVPA: moderate-vigorous physical activity
VO_2_: volume of oxygen
VO_2_max: maximal oxygen consumption
VO_2_R: oxygen consumption reserve
VO_2_rest: resting oxygen consumption
